# 4D Flow cardiovascular magnetic resonance consensus statement: 2023 update

**DOI:** 10.1186/s12968-023-00942-z

**Published:** 2023-07-20

**Authors:** Malenka M. Bissell, Francesca Raimondi, Lamia Ait Ali, Bradley D. Allen, Alex J. Barker, Ann Bolger, Nicholas Burris, Carl-Johan Carhäll, Jeremy D. Collins, Tino Ebbers, Christopher J. Francois, Alex Frydrychowicz, Pankaj Garg, Julia Geiger, Hojin Ha, Anja Hennemuth, Michael D. Hope, Albert Hsiao, Kevin Johnson, Sebastian Kozerke, Liliana E. Ma, Michael Markl, Duarte Martins, Marci Messina, Thekla H. Oechtering, Pim van Ooij, Cynthia Rigsby, Jose Rodriguez-Palomares, Arno A. W. Roest, Alejandro Roldán-Alzate, Susanne Schnell, Julio Sotelo, Matthias Stuber, Ali B. Syed, Johannes Töger, Rob van der Geest, Jos Westenberg, Liang Zhong, Yumin Zhong, Oliver Wieben, Petter Dyverfeldt

**Affiliations:** 1 Department of Biomedical Imaging Science, Leeds Institute of Cardiovascular and Metabolic Medicine (LICAMM), LIGHT Laboratories, Clarendon Way, University of Leeds, Leeds, LS2 9NL UK; 2 Children’s Hospital Meyer, University of Florence, Florence, Italy; 3 Institute of Clinical Physiology CNR, Massa, Italy; 4 Foundation CNR Tuscany Region G. Monasterio, Massa, Italy; 5 Department of Radiology, Northwestern University Feinberg School of Medicine, Chicago, IL USA; 6 Department of Radiology, Children’s Hospital Colorado, University of Colorado Anschutz Medical Center, Aurora, USA; 7 Department of Medicine, University of California, San Francisco, CA USA; 8 Department of Health, Medicine and Caring Sciences, Linköping University, Linköping, Sweden; 9 Department of Radiology, University of Michigan, Ann Arbor, USA; 10 Center for Medical Image Science and Visualization (CMIV), Linköping University, Linköping, Sweden; 11 Department of Radiology, Mayo Clinic, Rochester, MN USA; 12 Department of Radiology and Nuclear Medicine, University Hospital Schleswig-Holstein, Campus Lübeck and Universität Zu Lübeck, Lübeck, Germany; 13 Norwich Medical School, University of East Anglia, Norwich, UK; 14 Department of Diagnostic Imaging, University Children’s Hospital, Zurich, Switzerland; 15 Children’s Research Center, University Children’s Hospital Zurich, Zurich, Switzerland; 16 Department of Mechanical and Biomedical Engineering, Kangwon National University, Chuncheon, South Korea; 17 Institute of Computer-Assisted Cardiovascular Medicine, Charité – Universitätsmedizin, Berlin, Germany; 18 German Center for Cardiovascular Research (DZHK), Partner Site, Berlin, Germany; 19 Department of Diagnostic and Interventional Radiology and Nuclear Medicine, University Medical Center Hamburg-Eppendorf, Hamburg, Germany; 20 Department of Radiology and Biomedical Imaging, University of California, San Francisco, CA USA; 21 Department of Radiology, University of California, San Diego, CA USA; 22 Departments of Radiology and Medical Physics, University of Wisconsin, Madison, WI USA; 23 Institute for Biomedical Engineering, University and ETH Zurich, Zurich, Switzerland; 24 Department of Pediatric Cardiology, Hospital de Santa Cruz, Centro Hospitalar Lisboa Ocidental, Lisbon, Portugal; 25 Department of Radiology, Northwestern Medicine, Chicago, IL USA; 26 Department of Radiology & Nuclear Medicine, Amsterdam Cardiovascular Sciences, Amsterdam Movement Sciences, Amsterdam University Medical Centers, Location AMC, Amsterdam, The Netherlands; 27 Department of Pediatric Cardiology, Division of Pediatrics, Wilhelmina Children’s Hospital, University Medical Center Utrecht, Utrecht, The Netherlands; 28 Department of Medical Imaging, Ann & Robert H Lurie Children’s Hospital of Chicago, Chicago, IL USA; 29 Department of Cardiology, Hospital Universitari Vall d´Hebron,Vall d’Hebron Institut de Recerca (VHIR), Universitat Autònoma de Barcelona, Barcelona, Spain; 30 Centro de Investigación Biomédica en Red-CV, CIBER CV, Madrid, Spain; 31 Department of Pediatric Cardiology, Willem-Alexander’s Children Hospital, Leiden University Medical Center and Center for Congenital Heart Defects Amsterdam-Leiden, Leiden, The Netherlands; 32 Mechanical Engineering and Radiology, University of Wisconsin, Madison, WI USA; 33 Department of Medical Physics, Institute of Physics, University of Greifswald, Greifswald, Germany; 34 School of Biomedical Engineering, Universidad de Valparaíso, Valparaíso, Chile; 35 Biomedical Imaging Center, Pontificia Universidad Catolica de Chile, Santiago, Chile; 36 Millennium Institute for Intelligent Healthcare Engineering – iHEALTH, Santiago, Chile; 37 Département de Radiologie Médicale, Centre Hospitalier Universitaire Vaudois, Lausanne, Switzerland; 38 Department of Radiology, Stanford University, Stanford, CA USA; 39 Clinical Physiology, Department of Clinical Sciences Lund, Lund University, Skåne University Hospital, Lund, Sweden; 40 Division of Image Processing, Department of Radiology, Leiden University Medical Center, Leiden, The Netherlands; 41 CardioVascular Imaging Group (CVIG), Department of Radiology, Leiden University Medical Center, Leiden, The Netherlands; 42 National Heart Centre Singapore, Duke-NUS Medical School, National University of Singapore, Singapore, Singapore; 43 Department of Radiology, School of Medicine, Shanghai Children’s Medical Center Affiliated With Shanghai Jiao Tong University, Shanghai, People’s Republic of China

**Keywords:** 4D Flow CMR, 4D Flow MRI, Phase-contrast magnetic resonance imaging, MR flow imaging, Hemodynamics, Flow visualization, Flow quantification, Recommendations, Clinical, Cardiovascular, Heart disease

## Abstract

Hemodynamic assessment is an integral part of the diagnosis and management of cardiovascular disease. Four-dimensional cardiovascular magnetic resonance flow imaging (4D Flow CMR) allows comprehensive and accurate assessment of flow in a single acquisition. This consensus paper is an update from the 2015 ‘4D Flow CMR Consensus Statement’. We elaborate on 4D Flow CMR sequence options and imaging considerations. The document aims to assist centers starting out with 4D Flow CMR of the heart and great vessels with advice on acquisition parameters, post-processing workflows and integration into clinical practice. Furthermore, we define minimum quality assurance and validation standards for clinical centers. We also address the challenges faced in quality assurance and validation in the research setting. We also include a checklist for recommended publication standards, specifically for 4D Flow CMR. Finally, we discuss the current limitations and the future of 4D Flow CMR. This updated consensus paper will further facilitate widespread adoption of 4D Flow CMR in the clinical workflow across the globe and aid consistently high-quality publication standards.

## Introduction

This is an update to the 4D Flow CMR Consensus Statement published in 2015 [1].

Hemodynamics evaluation is crucial for the assessment of cardiovascular diseases, and is essential for understanding pathophysiology and explaining clinical manifestations. Four-dimensional cardiovascular magnetic resonance flow imaging (4D Flow CMR) uniquely provides comprehensive, in vivo characterization of cardiovascular blood flow. With this approach, the blood flow velocity is measured through motion encoding in all three spatial directions and resolved relative to all three dimensions of space and to the dimension of time along the cardiac cycle (3D + time = 4D).

4D Flow CMR is an extension of 2D Flow CMR [2–6] which is currently the most used clinical flow application. Visualization of flow direction and magnitude are also valuable in clinical practice. More advanced quantification parameters are to date still largely confined to the research arena.

Several review papers are now available on 4D Flow CMR [7–12], detailing its advantages over 2D Flow [13] as well as descriptions of useful clinical applications, especially for aortic disease [14–21], but also congenital heart diseases [22–28], particularly in the neonatal population [29], and other cardiovascular conditions [30–34]. We therefore will not be covering detailed benefits of 4D flow CMR and its clinical application in this consensus statement.

The previously published consensus statement [1] covers background information, clinical and scientific significance, and potential utility. Its recommendations regarding patient preparation, 4D Flow CMR data acquisition, data pre-processing, and flow visualization remain valid [1].

Since the publication of the original consensus statement, the field of 4D Flow CMR and the size of its user base has grown, supported by advances in CMR scanner hardware and coils, data acquisition and reconstruction strategies, vendor support, and availability of commercial post-processing solutions. Key advances in the last five years include further acceleration and diversification in acquisition methods. However, the most important development has been that 4D Flow CMR is now clinically available and supported by the major vendors of CMR scanners. Additionally, post-processing tools are commercially available, United States Food and Drug Administration (FDA) approved, European (CE-) marked for clinical use and in some countries, approved for reimbursement. These developments have enlarged the user base and paved the way for the more widespread clinical application of 4D Flow CMR, which is now used in the clinical routine at multiple centers worldwide. This is prompting large cohort, longitudinal and multi-center clinical studies. As the variety of acquisition and analysis platforms grows, standardized imaging acquisition, analysis, and publication approaches will simplify pooling data for meta-analysis studies and increase the validity of study results.

It is important to note that clinical and research 4D Flow CMR applications have differing priorities. Clinical acquisitions need to be fast with reliable flow and velocity quantification. In research, scan duration is less important whereas comprehensiveness of data is prioritized. Conversely, validation is more complex in the research setting as there often is no predefined gold standard for comparison for advanced measures beyond velocity and flow.

This update statement builds on the previously published consensus statement [1] and focuses on:

- Recommended acquisition parameters for clinical use—with a growing number of clinical centers starting out in 4D Flow CMR we have summarized updated clinical parameter recommendations based on consensus from centers clinically using 4D Flow CMR.
- Clinical post-processing workflow—this section describes key elements to consider and follow when choosing a clinical post-processing platform and setting up a clinical workflow.
- Quality assurance and validation advice—with a growing number of different sequences and post-processing platforms commercially available, we have further extended the advice for clinical quality assurance and validation when starting out with 4D Flow CMR. Furthermore, we address the challenges faced in quality assurance and validation in the research setting.
- Integration into clinical practice—this section covers a selection of advice from centers that have integrated 4D Flow CMR into the clinical workflow.
- Recommended publication standards—this section provides a checklist specific for 4D Flow CMR.
- Overcoming limitations and future considerations—focusing on what is on the horizon for 4D Flow CMR.
- Appendix: 4D Flow CMR sequence options—since the last consensus statement, the options of available 4D Flow CMR sequences have considerably increased. This appendix, therefore, summarizes some aspects to consider when choosing a sequence prior to setting up a research study or clinical service.

This consensus update is based on published data, where available, and consensus experience. It aims to cover a large audience, including clinicians and scientists interested in starting out in 4D Flow CMR as well as bringing together established groups in the area.

## Advised acquisition parameters for clinical use

The choice of 4D Flow CMR acquisition parameters requires careful consideration of a balance between accuracy and scan time. For clinical use, it is advisable to keep the 4D Flow CMR acquisition to 5–10 min. Thereby it can easily be added to a clinical workflow, such as in the waiting period between gadolinium administration late enhancement enhancement (LGE) imaging, without interfering with or extensively prolonging the established protocols in the institution.

### Equipment and set-up

The improved signal to noise ratio (SNR) at higher field strength such as 3T can be beneficial in the younger pediatric setting given the higher spatial resolution needed due to the small body size anatomy but is less important in older children and adults where body size is sufficient for good SNR at lower field strengths.

Coil selection largely depends on local protocol and availability. The routine number of coil elements used in standard cardiac imaging is sufficient for good quality 4D Flow CMR acquisition. The number of coil elements needs to be balanced against the ability of the scanner to reconstruct the data in a timely manner.

Volume coverage ideally includes at least the valves and aortic and pulmonary sinuses (even if focusing on intra-cardiac anatomy) for data quality assurance purposes (see section “Quality assurance and validation advice for clinical use”). Appropriate field-of-view that fully covers the anatomy of interest (plus a couple of additional slices) can be confirmed using anatomical scout images on the scanner.

For ease of use, it is best to aim for standardized protocols which might need to be adjusted for individual pathologies. Congenital heart disease centers in particular might have a variety of protocols for different age groups and/or pathologies. These should all be individually validated (see section “Quality assurance and validation advice for clinical use”).

### Scan parameters

Accuracy and precision in flow imaging are influenced by several physiological patient parameters. Different body sizes and heart rates influence spatial and temporal resolutions, SNR, and therefore velocity-to-noise ratio (VNR) [35, 36]. Hence, we recommend adjusting the spatial resolution for different age groups. Voxels should be isotropic and at least 6 voxels should cover a vessel diameter [37]. Specific resolution guidance based on the most common resolutions used in clinical centers are detailed in Table 1.

**Table 1:** 4D flow acquisition parameters for large vessels and whole heart

Acquisition parameter	Aim for	Do not exceed	Reason	Limiting factor
*Acquired spatial resolution*				
Adults whole heart	2.5 mm^3^ [2]	3 mm^3^	Accuracy	Scan time
Adults’ vessels	2 mm^3^	2.5 mm^3^	Accuracy	Scan time
Pediatric whole heart	2 mm^3^ [53, 79, 82]	2.5 mm^3^ [82, 167]	Accuracy	Scan time
Pediatric vessels	1.5 mm^3^	2 mm^3^	Accuracy	Scan time, VNR
Neonates	0.75–1 mm^3^ [54]	1.5 mm^3^	Accuracy	Scan time, VNR
Acquired temporal resolution	30 ms	50 ms (can be higher if aiming for visualization only)	Accuracy	Scan time
Velocity encoding limit (VENC)	Maximum expected velocity	10% higher than maximum expected velocity, do not exceed 25%	VNR, to avoid aliasing	Scan time, VNR
ECG gating	Retrospective		Complete ECG cycle included	Reconstruction
Respiratory navigation	Optional		Accuracy	Scan time
Contrast agent	Consider in neonates, dissection patients or other challenging cases		Improved contrast	Contrast administration contraindications
Flip angle	7° non-contrast 12–25° with contrast	25°	SNR	Contrast vs SNR

It is important to note here the difference between acquired resolution, based on the field-of-view and k-space matrix size, and the reconstructed resolution, which is often higher than the acquired due to the use of spatial interpolation during image reconstruction. This is an important distinction, since sequence performance is primarily defined by the acquired rather than the reconstructed resolution. For completeness and easier comparison, both acquired and reconstructed resolutions should be stated in scientific publications.

4D Flow CMR requires the selection of an upper velocity encoding limit (VENC) during scan prescription to avoid velocity aliasing. This setting will adjust the motion encoding gradients accordingly to the desired motion sensitivity. In non-contrast acquisitions, the VENC should be as low as possible to keep adequate VNR and improve accuracy while avoiding aliasing [36].

Choice of VENC should be close to the maximum velocity (< 25% above) [38] and can be guided by a previous imaging examination when available, such as a recently acquired echocardiogram or a previous CMR study. Otherwise, a 2D phase-contrast acquisition or rapid velocity scout sequence can be used at the aortic valve or area of interest. If 4D Flow CMR is acquired without a previous 2D phase-contrast acquisition and stenosis is suspected, consider an initial VENC at 250 cm/s. If no stenosis is suspected, the following VENC can be used as guidance:

- Large vessels (pulmonary artery and aorta): 150 cm/s [25]
- Dissection false lumen: 50–150 cm/s
- Venous blood flow (including extracardiac conduit and pulmonary arteries in Fontan patients): 50–80 cm/s
- Intra-cardiac: 100–150 cm/s

Electrocardiographic (ECG) gating should be retrospective whenever possible to capture hemodynamics throughout the complete cardiac cycle [39–42]. Operators should monitor the ECG signal and acquisition time estimates to determine if electrodes require repositioning, as poor ECG signals can lead to prolonged scans and reduced image quality and accuracy. Irregular heartbeats can be a challenge, but 4D Flow CMR acquisition can still be accurate in patients with atrial fibrillation [43] but is not always reliable. Prospective gating and arrhythmia rejection might be useful in these cases.

Respiratory motion suppression can improve image quality [44, 45] and does not automatically increase scan time, and guidance should be available from vendors or sequence developers whether respiratory motion suppression is advised for the specific sequence. In practice many clinical centres do not use respiratory motion suppression as it is not available from all vendors for all sequences. Furthermore, in patients with fast heart rates (HR; such as neonates or during stress or exercise with HR > 120 bpm) diaphragmatic respiratory navigators are often not feasible. If respiratory suppression is desirable, other respiratory gating methods such as self-gating or respiratory bellows can be considered. When self-gating becomes readily available, this would be the recommended gating method.

### Contrast agent and flip angle

Advised scan parameters are summarized in Table 1.

As many acquisition parameters are likely to influence 4D Flow CMR acquisition, it is important to have consistent protocols which have undergone local quality assurance testing.

The spoiled gradient-echo sequence with short repetition time (TR) generates phase-contrast angiograms without the need for contrast agents [46, 47]. SNR and VNR improve with T1 shortening achieved by administering gadolinium-based contrast [48, 49] or superparamagnetic iron oxide agent (ferumoxytol) [50]. Therefore, contrast administration to enhance image quality can be useful (but is not essential), especially in challenging cases such as neonates and dissections to enhance image quality, but good image quality can be achieved without contrast administration especially when scanning at 3T [51, 52]. In adults, if contrast is given for other reasons, it is useful to perform the 4D Flow CMR acquisition after gadolinium-based contrast administration. It is important to note, that contrast administration for 4D Flow CMR acquisition alone is not required especially as the wider CMR community is moving more towards non-contrast acquisitions.

The standard flip angle for non-contrast 4D Flow CMR acquisition should be set around the Ernst angle which equates to a flip angle of around 7° for non-contrast 4D Flow CMR with repetition time and echo time chosen as short as possible. After contrast administration a higher flip angle is often beneficial, but this depends on contrast agent used and time past since administration. Several clinical centres use the following guidance: If acquiring 4D Flow CMR directly after gadolinium administration, it is advisable to increase the flip angle to 15–25° 1.5T and 12° at 3T. [29, 49, 53]. If 4D Flow CMR is acquired after LGE a lower flip angle (similar to non-contrast values) is likely needed. If using ferumoxytol a higher flip angle of around 15–25° is often required [54, 55]. In neonates, limits in specific absorption rate (SAR) often necessitate dropping the flip angle with ferumoxytol to 12° in this patient cohort [54].

## Clinical post-processing workflow

**Fig. 1:**
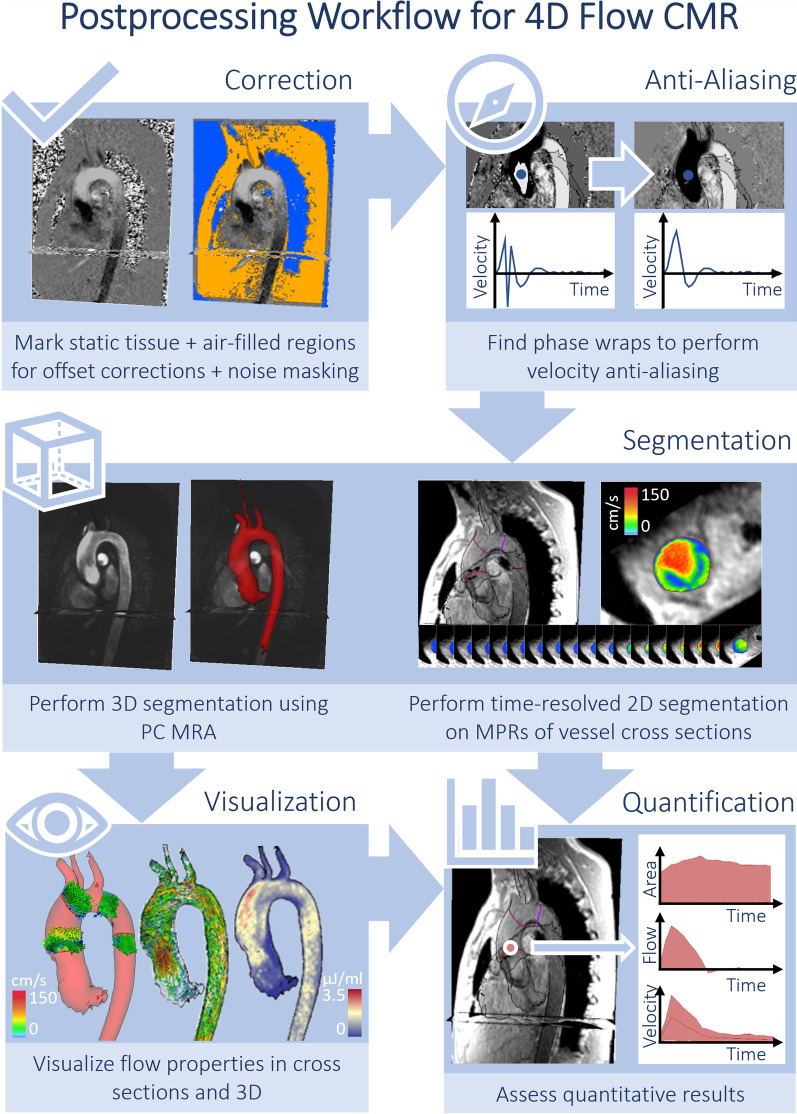
Post-processing of 4D Flow CMR should always include correction for phase offsets and noise masking. Anti-aliasing needs to be performed if aliasing is present in regions of interest. Segmentation can be performed for the whole vessel in 3D or on 2D vessel cross-sections perpendicular to the course of the vessel. Visualization of flow, velocity and advanced parameters is optional but can help identify regions of peak velocities and insufficiencies. Quantification can be performed in 2D cross sections or in regions of the vessel. Parameters can be given averaged over the whole cardiac cycle (e.g. stroke volume) or maximum and minimum parameters (e.g. peak velocity)

Data pre-processing steps are described in detail by the previous 4D Flow CMR consensus statement and this remains valid [1]. Key elements are summarized in Fig. 1.

Post-processing of 4D Flow CMR data includes the following steps: (1) background phase offset correction, (2) anti-aliasing if required, (3) segmentation, (4) visualization (optional), and (5) quantification including an internal consistency check (Fig. 1).

There are several commercially available software packages for post-processing and analysis of 4D Flow CMR. Most software packages have regulatory approval for basic flow quantification in clinical routine. In addition, they allow visualization of blood flow and the analysis of various advanced research parameters such as flow eccentricity, vortices, kinetic energy (KE), flow components as well as relative pressure distribution for research purposes. Many software packages now also include valve tracking, circumventing the issue of through-plane motion and permitting valve motion to be factored in when computing flow, which improves accuracy especially in mitral and tricuspid valve assessment [56].

### Step 1: Background phase offset correction

For accurate flow measurements, 4D Flow CMR requires correction for phase offset errors associated with eddy currents and concomitant gradient fields if not corrected during image reconstruction. While offset errors can be corrected by repeating the exam with a stationary phantom and subtracting the flow measurements of the static tissue from the patient’s data [57], this is too time-consuming for clinical practice. Static-tissue interpolation offset correction can be applied during post-processing with equivalent performance [37, 58]. All software should have this capability using linear or polynomial fits to static tissue. Particular attention should be given to large fields-of-view where regions-of-interest may reside far away from the magnet isocenter as offset errors increase with distance from the magnet isocenter [59].

### Step 2: Velocity anti-aliasing

In cases where maximum blood flow velocity surpasses the chosen VENC, velocity aliasing can result in corrupted velocity measurements. In these cases, phase unwrapping can improve the accuracy of the flow and velocity measurements [60–65]. Most software can detect a large shift in adjacent voxel velocity values and perform automatic correction. However, visual inspection of the peak systolic and diastolic cardiac phases is required to check all three primary velocity encoding directions for un-correctable velocity aliasing. Image regions affected by incorrigible aliasing, should not be considered for flow analysis.

### Step 3: Segmentation

Depending on the software solution, evaluation of flow data starts either with 3D segmentation of the vessel or direct placement of regions of interest in the imaging volume delineating the vessel contour in 2D cross-sectional planes. Appropriate regions of interest selection, orientation, and segmentation are important parts of the flow and velocity quantification process [66]. Care must be taken to select regions unaffected by artefacts, e.g., caused by partial volumes, metal implants, or motion. Vascular flow values should be measured in 2D planes that are orthogonal to the vessel. Regions of interest need to be propagated and adjusted throughout the cardiac cycle to account for vessel motion. Centerline-based plane positioning and registration-based contour propagation can support this process.

#### Valve tracking

**Fig. 2:**
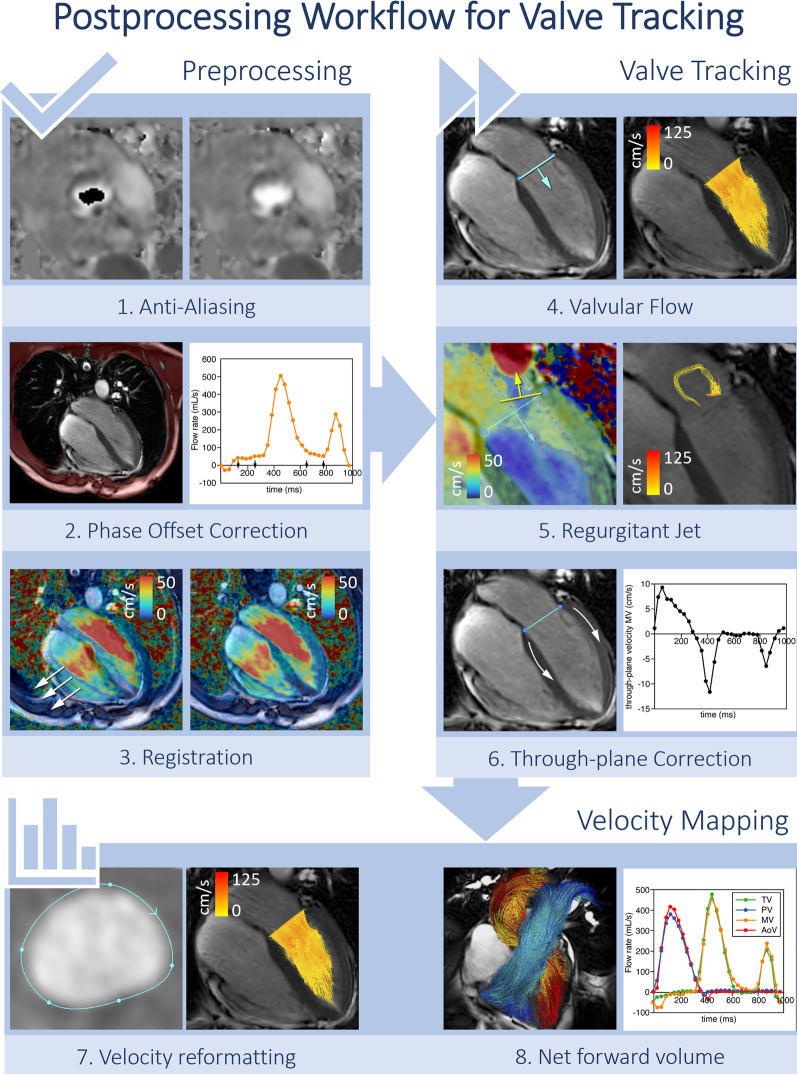
Valve tracking procedure in 4D flow CMR. In preprocessing phase, velocity data is corrected for aliasing (1), phase offset correction (2) and misregistration (3). Annulus tracking (4) is performed for forward flow and backward flow is obtained by tracking the regurgitant jet (5). Velocity corrections are performed by subtracting through-plane valve motion (6). Then, velocity mapping is performed on the reformatted 2D through-plane velocity images (7). Finally, the net forward volume among the four valves can be used as an internal check for consistency in the analysis (8)

Using retrospective valve tracking, a dynamic reformatted 2D plane of through-plane velocity is created from the time-resolved 3D velocity data (Fig. 2). Two orthogonal cine views per valve (for instance, left ventricular (LV) two-chamber and four-chamber views for the mitral valve) should be used to track the valve annulus over the whole cardiac cycle. Misalignment between the cine views and the 4D Flow CMR data should be resolved by manual or automatic image registration. It is advised to quantify regurgitant jets separately, by defining a reformatted plane perpendicular to the regurgitant jet [67]. Aliasing in the regurgitant jet is common, as regurgitant flow is usually characterized by high blood velocity, turbulence, and incoherent flow and in these cases indirect quantification is advised (see section “Step 5: Quantification” below). The regurgitant jet regions of interest should be segmented and propagated in the reformatted 2D plane as described above (see Fig. 2).

### Step 4: Visualization

Visualization can be performed using multiple tools such as velocity-based color coding, maximum velocity projections (“velocity MIP”), instantaneous streamlines, and time-resolved pathlines. The differences between streamlines and pathlines are described in detail in the first 4D Flow CMR Consensus Statement [1]. We emphasize that streamlines do not represent flow pathways in pulsatile blood flow; keeping streamlines short minimizes the risk for misinterpretation. Visualization should include dynamic visualization of the complete 3D volume as well as localized visualization tools for the particular region of interest [68]. Visualization can serve as a quick quality assessment in cases where velocity values are inverted. Visualization facilitates the detection and understanding of blood flow alterations in different pathologies, such as shunts or valve insufficiencies. Further detailed background information of 4D flow CMR visualization can be found in the 2015 consensus statement [1].

### Step 5: Quantification

Guided by the visualization of anatomy and blood flow, 2D planes can be placed to measure flow parameters at anatomical landmarks or in areas of pathological flow. The most relevant clinical 4D Flow CMR-derived parameters are flow volumes and flow velocities that should be provided in the clinical report. Quantitative data should always be validated for internal consistency (see section “Quality assurance and validation advice for clinical use”).

The accuracy of blood flow can be compromised in certain flow geometries, and readers should be cautious of flow measurements in areas of high velocity flow jets, regions with substantial dephasing due to turbulence, and highly vortical blood flow [69], especially in ascending aortic aneurysm or aneurysmal pulmonary arteries. In these circumstances, alternative flow measurements outside of regions of abnormal flow or combined use of ventricular volumetry may be necessary to guide clinical management [70]. Examples include using superior vena cava and descending aortic flow as net forward flow in the aorta. For evaluation of valvular regurgitant volume both direct and indirect jet quantification methods are used [71].

#### Direct jet quantification

The direct jet tracking method should be used in regurgitant lesions with only one central jet such as aortic and pulmonary regurgitation, in functional mitral regurgitation [72] or atrioventricular valve regurgitation after atrioventricular septal defect correction [73]. The advantage of this direct measurement approach is that no assumptions are made with respect to regurgitant jet morphology or mass conservation through other valves or over the atrial or ventricular septum, and that flow quantification over all four valves is performed from the same dataset from the same average cardiac cycle.

#### Indirect quantification method

The standard CMR method for mitral regurgitation quantification, here called *indirect quantification,* involves the subtraction of the aortic net LV ventricular stroke volume (SV) determined by LV cine short-axis volumetric assessment. Valve tracking has led to the improved indirect method involving subtraction of aortic net flow from the mitral forward flow. In cases where there are multiple jets with different directions or the regurgitation jet has uncorrectable aliasing, we recommend using the indirect method with valve tracking through the mitral and aortic valves as this has been shown to be more accurate in these cases [74, 75].

While using any 4D Flow CMR method for assessment of valvular regurgitation, it is recommended to cross-check the quantification against standard methods. If there is a significant discrepancy in the quantification of regurgitation volume between methods (> 15 ml or > 10%), it is recommended to revisit the analysis and investigate the cause of the discrepancy using the conservation of mass principle (i.e. flow into and out of a chamber should be balanced). The following equations can be used to check the consistency of flow data:

LV stroke volume (short-axis cine segmentation) = mitral forward flow + aortic backward flow = aortic forward flow + mitral backward flow.

Retrospective valve tracking can be applied in patients with atrial fibrillation [76]. However, a cautious approach should be used as there is a possibility of underestimating flows. In these cases, relative flow quantification, for example, regurgitation fraction, is possibly more reliable than absolute numbers of regurgitation volume.

## Quality assurance and validation advice for clinical use

Both initial validation and ongoing quality assurance are important aspects of clinical 4D Flow CMR [77]. This section builds and expands on the 2015 consensus statement [1].

For incorporation into standard clinical practice, 4D Flow CMR acquisitions must meet quality thresholds that provide the interpreting clinician with confidence in both the qualitative and quantitative accuracy of the data. Accuracy in 4D Flow CMR can be influenced by the choice of vendor sequences [77], acquisition parameters [2] and postprocessing software [78]. Therefore, local validation and ongoing quality assurance are an important part of the clinical 4D Flow CMR workflow.

**Initial validation** is advised to be undertaken when using a new sequence, updating a sequence (such as a significant sequence change with system updates), gradient servicing, applying significant changes in acquisition parameters or using a new post-processing platform:

We advise acquiring 10 datasets (healthy subjects and/or patients without any intra- or extra-cardiac shunts) with both the institution’s standard 2D Flow and 4D Flow CMR including at least: ascending aorta, pulmonary trunk, left branch pulmonary artery, right branch pulmonary artery, superior vena cava, descending aorta and pulmonary veins. If possible we also advise to rescan the volunteers/patients ideally after exiting and then re-entering the scanner either on the same day or a defined short recall period (< 1 month).

Initial visual assessment of the velocity and magnitude images should include assessment of motion artefacts, wrap around artefacts and any aliasing in systole.

We advise to include 3 steps in the quantitative assessment: (1) comparison to 2D Flow CMR, (2) within dataset validation, (3) inter- and intra-reader comparison (when changing/updating post-processing platform). Ideally, differences in flow assessment should be ≤ 5%. Scan-rescan differences up to 10% are acceptable due to minor physiological differences between scans.

**Fig. 3:**
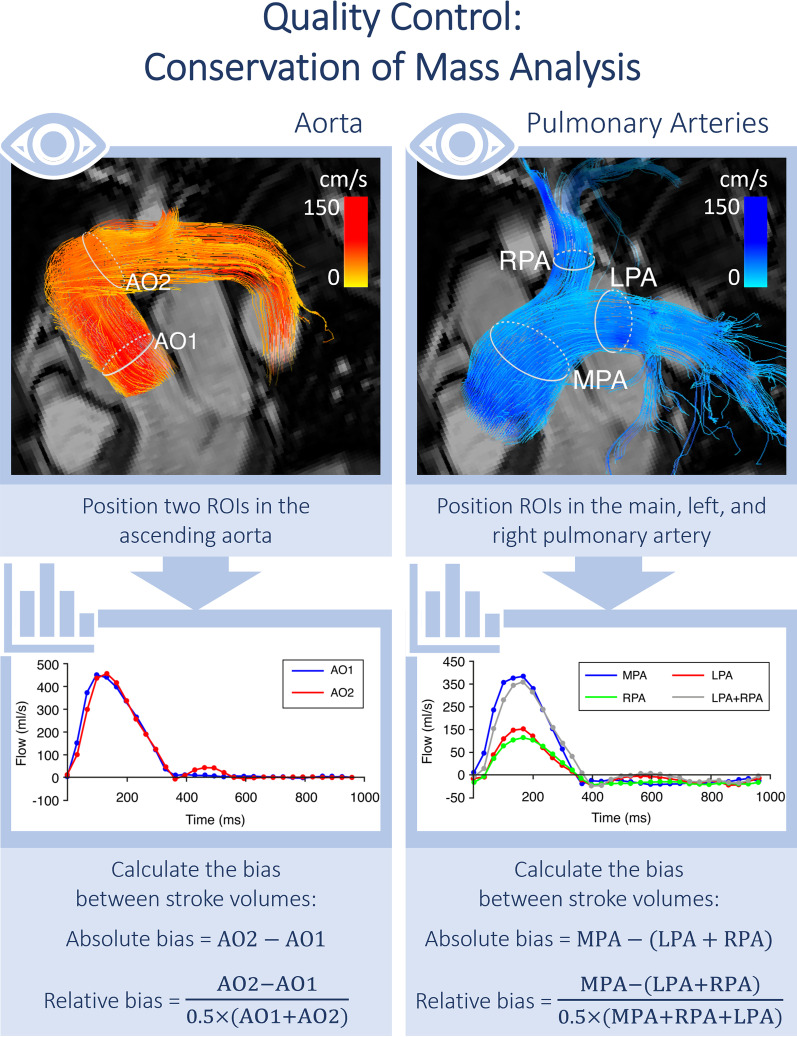
Internal consistency of measurements can be checked by comparing flow volumes at different locations in the same vessel or by comparing the sum of branch vessels to the main pulmonary artery

Comparison to 2D Flow CMR: We suggest comparing forward flow and peak velocity for at least ascending aorta, pulmonary trunk, left branch pulmonary artery, right branch pulmonary artery, superior vena cava and descending aorta between 2D Flow and 4D Flow CMR in the 10 validation datasets. This captures arterial and venous flow with different flow velocities as well as a variety of vessel diameters (see Fig. 3).

Within dataset validation: This makes use of the conservation of mass principle. Mass is neither created nor destroyed and so flow volumes should stay equal. Therefore, the following forward flow comparisons can be made in all 10 datasets and should show equal flow:

- Aortic flow (add 5% for coronary flow if measuring above sinuses) = pulmonary flow
- Right + left pulmonary artery flow = main pulmonary artery flow
- Branch pulmonary artery flow = pulmonary vein flow (If not equal check for pulmonary vein anomalies)
- Superior vena cava + descending aortic flow = ascending aortic flow

This allows the assessment of measurement planes in a variety of directions within the 4D Flow CMR dataset.

Furthermore, we advise placing 2–4 measurement planes in the ascending aorta between the sinuses of Valsalva and the 1st branching vessel. Again, using the conservation of mass principle, the flow volume should match in all planes (< 5% difference).

Inter- and intra-reader comparison: We advise each reader involved in the clinical service to complete the above flow validations in all 10 datasets twice at least 1 week apart to evaluate any inter or intra-reader bias. Differences in flow assessment should be ≤ 5%.

**Everyday quality assurance** in every dataset acquired should at least include:

Initial visual assessment of the phase contrast and magnitude datasets should include assessment of motion artefacts, wrap around artefacts and any aliasing during systole.

Quantitative assessment using within dataset validation using the conservation of mass principle. At least one of the above ‘within dataset’ forward flow comparisons can be completed. The choice of these depends on the underlying anatomy and physiology and is determined by the reading physician.

## Integration into clinical practice

### Considerations for integration into clinical practice

When embarking on integrating 4D Flow CMR into the clinical workflow, several considerations are important. Initial validation of the chosen 4D Flow sequence on the local CMR scanner is paramount (see section "Quality assurance and validation advice for clinical use"). 4D Flow CMR datasets are large and may require additional space on the hospital’s image storage solutions. Stored datasets need to be accessible by 4D Flow CMR analysis software which often requires integrated graphics processing units (GPUs) and higher processor powers than standard hospital computers. An alternative is cloud-based 4D Flow CMR offered by some software vendors.

As with any new imaging technique, it takes a while for the teams involved to get confident with image acquisition and analysis [79]. Only when this is achieved should the data be used for clinical reporting. Initially, both 2D and 4D Flow CMR should be acquired and analyzed in parallel. All published prognostic values are based on 2D Flow CMR and the clinical team will need time to evaluate whether 4D Flow CMR assessment can be used interchangeably with 2D Flow CMR in all or some of their patient cohorts.

### Where does 4D Flow CMR fit in a clinical protocol?

Historically, 4D Flow CMR has been considered a research technique and thus placed at the end of clinical exams after diagnostic sequences; however, with the emergence of clinically validated applications and postprocessing software, some centers are now adding 4D Flow CMR to routine clinical CMR protocols [79–82]. When deciding where to place 4D Flow CMR within a clinical CMR protocol there are several considerations, which take into account scan time and pathology-specific considerations:

1. Non-contrast 4D Flow CMR is sufficient in many scenarios. If administering gadolinium-based contrast agents for other clinical questions, 4D Flow CMR should be placed after the CMR angiogram or during the delay before myocardial LGE. It is important to note that technical factors such as respiratory compensation, multi-VENC and large field-of-view increase scan time and may prohibit 4D Flow CMR acquisition during the 10-min post-gadolinium delay window.
2. If the clinical indication requires flow quantification (e.g., shunt evaluation, quantification of valvular regurgitation) then 4D Flow CMR may take higher priority and be acquired earlier in the imaging protocol, especially if 4D Flow CMR is used in place of standard 2D Flow imaging.
3. In pathologies where 4D Flow CMR plays an adjunctive role (e.g., aortic aneurysm), ensuring that all diagnostic sequences are completed before the acquisition of 4D Flow CMR is a common approach

### Quantitative analysis

Quantitative analysis of flow volumes and peak velocity can easily be integrated into standard CMR reporting templates. We advise reporting both 2D and 4D Flow CMR concomitantly until both imaging and clinical cardiology teams feel confident in basing clinical decisions on 4D Flow CMR results alone.

More advanced parameters can be derived from 4D Flow CMR velocity maps using dedicated software, but these may not be formally approved yet for clinical use.

### Qualitative analysis

To take full advantage of the comprehensive nature of 4D Flow CMR, users should interact with the 4D Flow CMR datasets that can be reformatted into any plane to derive the optimum qualitative patient data for display. This is not possible on standard viewing and analysis platforms accessible to clinicians using images for decision making. We, therefore, recommend developing a workflow to save useful 4D Flow CMR images/videos in DICOM format which can then be loaded onto standard viewing platforms. This makes 4D Flow CMR more accessible to clinicians and can be used at multi-disciplinary team meetings even without a 4D Flow CMR specialist present. Especially in congenital heart disease qualitative analysis can be useful in delineating stenosed vessels in more detail, providing information on the exact position and length of flow acceleration. It also easily identifies any flow reversal.

## Quality assurance and validation advice in the research setting

Several useful options exist for the validation of different aspects of 4D Flow CMR, such as sequence development, reconstruction algorithms and post-processing workflows. These options can broadly be categorized into (1) in vivo studies, (2) phantom studies, and (3) computer simulations (summarized in Table 2). We emphasize that there is no single evaluation or validation methodology that can target all aspects of 4D Flow CMR. Instead, evaluation and validation need to be tailored for the specific sequence, parameter, or application in question.

**Table 2:** Comparison of 4D Flow MRI Validation Methods

Validation method	Advantages	Disadvantages	When to use (examples)	Examples
In-vivo	Fidelity with respect to clinical or research use of the method	Physiological variability, commonly a lack of gold standard	Verification that method works in vivo	[83–86]
Phantoms	Controllable, easier to get a gold standard than in-vivo studies, even simple experiments can be of value	Realistic models challenging to construct and control	Evaluation variables/parameters in a repeatable setting in real MRI hardware	[87–91, 168]
Simulations	Very controllable, different sources of error can be separated, underlying numerical velocity data serves as ground truth	Fidelity uncertain, computational cost	Rapid feedback during development, when the desired evaluation cannot be realized in a phantom setup or in vivo	[92–94, 169]

### In vivo studies

In vivo studies are used to evaluate and validate new 4D Flow CMR methods in comparison to other modalities such as echocardiography, 2D Flow CMR, and other 4D Flow CMR methods [83–85]. Furthermore, it is possible to use consistency criteria such as conservation of mass principles, given the fact that flow into and out of a closed system (e.g., the heart, or the aorta) must be the same [85, 86]. The main advantage of using in vivo studies for evaluation and validation is that it represents the final utility of the method. A challenging aspect of in vivo studies is that reference data is often not available when advanced hemodynamic parameters such as wall shear stress, turbulence stresses, intracardiac flow component, kinetic energy and vorticity are evaluated. Furthermore, we emphasize that in vivo validation of the capability of a sequence to measure basic parameters such as flow volume cannot be interpreted as evidence that the sequence permits accurate estimation of advanced hemodynamic parameters, and these require separate and targeted validation.

### In vitro studies

In vitro phantom studies in idealized or anatomically accurate vascular and cardiac models, so-called flow phantoms, permit evaluation and validation in well-known and repeatable flow conditions. An advantage of in-vitro phantoms is that long sessions with scans using many different parameter settings can be performed on the same flow setup. Another advantage is the possibility to validate post-processing software and compare the results against flow meter, “timer and beaker” and pressure probe measurements, as well as other experimental fluid dynamics techniques, such as particle image velocimetry and direct pressure measurements [87–89]. A disadvantage is that in vitro phantoms typically do not have realistic surrounding tissue. Furthermore, we note that while advanced flow phantoms with realistic geometry and pulsating flow are highly valuable, even simplified phantom experiments can provide valuable insight. Examples include a large container of stationary water or agarose gel for the evaluation of background phase offsets and rotating phantoms consisting of gel-filled wheels or rings [90, 91]. Finally, we encourage the continuing development of standardized 4D Flow CMR phantoms and pump setups that facilitates reproducible in-vitro flow experiments across multiple sites.

### Computer simulations

Simulated 4D Flow CMR measurments in numerical velocity data, also referred to as synthetic phantoms and digital reference objects, permit detailed studies of the impact of sequence design and parameter settings in a fully known flow environment [92–95]. Another advantage of this approach is that synthetic phantoms can be created with a model of more realistic surrounding tissue. This is relevant for reconstruction algorithms and processing tools. Hybrid in vitro/synthetic phantoms in which in vitro data are embedded into synthetic backgrounds may also be considered. The main disadvantage of synthetic phantoms is the question of simulation fidelity, i.e., how well the simulation results represent reality. Increased fidelity usually requires more computational resources. It can therefore be relevant to consider how complete the simulation needs to be and if the aim of the study permits any trade-offs between simulation time and completeness of the 4D Flow CMR simulations. We note that the generation of accurate numerical velocity data using computational fluid dynamics is a separate field of research. However, when used as input and reference in 4D Flow CMR simulations, the physical accuracy of the numerical velocity data is of secondary importance.

In summary, the development of 4D Flow CMR methods can be guided by in vivo studies, phantom studies, computer simulations, or a combination thereof. We recommend that all these approaches be considered in the evaluation and validation of new developments in 4D Flow CMR.

## Recommended publication standards

In this section, we describe the essential and recommended standards that should be adhered to for any scientific publication containing 4D Flow CMR. Recommendations differ in parts between technical publications (often aiming to propose and evaluate a new technique) and clinical studies (applying 4D Flow CMR to clinical questions). High-quality and standardized publications will enable easier replication of proposed sequence protocols for clinical use and facilitate easier and higher quality meta-analysis to move the field forward.

Where possible, sharing of published datasets, code and materials to replicate, verify and extend the research presented in the manuscript is encouraged.

Below are 4D flow CMR specific considerations:

### Introduction

For studies based on a priori stated hypotheses, all hypotheses should be clearly stated when describing the aim(s) of the study.

### Methods-acquisition

All data processing methods that can affect the quality of the 4D flow CMR data should be described, including correction methods for eddy currents, distortions resulting from gradient field non-uniformity, intravoxel dephasing and concomitant gradient fields, velocity aliasing, as well as noise filtering (if not using commercially available sequences and data processing software). Many parameters, including hardware specifics, acquisition parameters, and post-processing software, affect the image quality and properties of 4D Flow CMR data and should be reported as such. The essential and recommended standards are listed in Table 3 and elucidated below. We specifically highlight that both acquired and reconstructed resolutions should be given, both for temporal and spatial resolution.

**Table 3:** Essential and recommended standards to be reported in a scientific publication containing 4D Flow MRI data

	Explanation	Technical studies	Clinical studies	Example
Hard- and software				
Contrast agent administration	If contrast agent used: Which contrast agent, dose, and timing in relation to 4D Flow CMR	Essential, if contrast agent used	Essential, if contrast agent used	gadolinium contrast agent (*insert name here*) was injected prior to the data acquisition
Manufacturer	Manufacturer of MRI system	Essential	Essential	GE, Philips, Siemens, etc
Scanner software version	Version of the scanner software during the study	Essential	Recommended	R11.2, VE11A
Field strength	Field strength in Tesla	Essential	Essential	1.5T
4D Flow CMR sequence	Description (clinical or research, through vendor, through academic partnership, in-house developed)	Essential	Essential	WIP
Coil type and channels	Coil types, number of elements	Essential	Recommended	16 channel cardiac coil
Acquisition parameters				
Motion encoding scheme	4, 5, 7 or 8-point encoding, variable venc encoding or other multi-venc approaches, as well as flow compensation versus two-point encoding	Essential	Recommended	[Example]
k-space filling method	Type of k-space acquisition such as cartesian, radial, spiral, pseudo-random, EPI, etc	Essential	Essential	Cartesian k-space filling
Acquired spatial resolution	Resolution of the acquired data before Fourier transform (FOV/matrix size)	Essential	Essential	2.5 × 2.5 × 2.5 mm^3^
Reconstructed spatial resolution	Spatial resolution of the reconstructed data (often interpolated during reconstruction)	Essential	Recommended	1.0 × 1.0 × 1.0 mm^3^
Effective temporal resolution	Typically: TR x flow encodings x # segments	Essential	Essential	35 ms
Reconstructed number of timeframes	Number of timeframes after reconstruction (often interpolated during reconstruction)	Recommended	Recommended	30
Echo time (TE)	Time between RF excitation and echo	Essential	Essential	2.6 ms
Repetition time (TR)	Time between to subsequent RF excitations	Essential	Essential	5.2 ms
Flip angle (FA)	Flip angle	Essential	Essential	5
Velocity encoding limit (VENC)	Maximum VENC	Essential	Essential	120 cm/s
Cardiac gating	Prospective triggering or retrospective cardiac gating, ECG, pulse oximeter or other devices	Essential	Essential	retrospective vector cardiogram controlled cardiac gating
Respiratory motion suppression	If respiratory motion suppression is performed	Essential	Essential	Navigator width (*insert here*) or acceptance window (*insert here*)
Acceleration method and factor	Acceleration approach used, such SENSE, GRAPPA, compressed SENSE, etc.)	Essential	Essential	parallel imaging (SENSE) speed up factors = 3 (AP direction), 1.6 (RL direction)
Typical scan time	Scan time assuming a regular heart rhythm of 60 bpm and regular breathing pattern or the average and range of the scan time of all subjects	Essential	Essential	10:30 min
Field of view (FOV)	Important to choose correctly to eliminate wrap; impacts spatial resolution	Recommended	Recommended	250.1 ± 10.2 × 280.1 ± 15.2 × 70.1 ± 10.8 mm^3^
Matrix size	Impacts spatial resolution	Recommended	Recommended	
Slab orientation	Orientation of the FOV	Essential	Essential	sagittal oblique orientation
Phase-encoding direction	Direction of the phase-encoding direction (were wrap around effects can be expected)	Recommended	Recommended	Phase-encoding direction in the inferior-superior direction
k-space segmentation factor	Number of k-space lines acquired per heartbeat	Essential	Recommended	2
Postprocessing				
Analysis software	Description (clinical or research, through vendor, through academic partnership, in-house developed, open-source availability)	Essential	Essential	In-house developed, availability at Github
Background phase offset corrections	Eddy currents, concomitant gradient fields, and distortions	Essential	Recommended	corrected for concomitant gradient field and eddy currents induced offset using a 2nd order correction
Velocity aliasing correction (phase unwrapping)	Description of velocity anti-aliasing algorithm, sharing of results if available	Essential	Essential	correction for velocity aliasing using a 4D Laplacian phase unwrapping (ref, github)
Segmentation method	Description of segmentation method used, if used for quantification and/or segmentation (Systolic segmentation, or time-resolved segmentation. Based on absolute velocity, magnitude weighting for each time frame and sum of squares, temporal absolute velocity average with magnitude weighting, piecewise pseudo complex difference, atlas-bases segmentation, AI based segmentation)	Essential, if used	Essential, if used	delineation of the aorta based on piecewise pseudo complex difference PC-MRA
Quantification				
Analysis software	Description (clinical or research, through vendor, through academic partnership, in-house developed, open-source availability)	Essential	Essential	Clinical analysis software package (*insert name here*)
Plane-wise analysis (peak velocity, flow rate, regurgitation volume)	Description of the plane positioning method (Manual, automatic, Fixed or valve tracking)	Essential	Essential	A 2D plane was manually located at the aortic valve at a fixed/valve tracking location over the cardiac cycle
Volumetric analysis (peak velocity, wall shear stress, turbulent kinetic energy, flow component analysis)	Description of the segmentation method (Manual, automatic, semi-automatic, Static or varying over the cardiac cycle)	Essential	Essential	The heart chambers and large vessels were segmented automatically over the cardiac cycle using AI (ref)
Gradient calculations (wall shear stress, viscous energy loss, pressure difference)	Description of the numerical difference method (Forward-, backward-, central-difference 1st order or higher order differences, Finite element method)	Essential	Recommended	2nd order central difference
Results				
Data quality assessment	Results of the data quality assessment both visual and quantitative such as conservation of mass error	Essential	Essential	Data quality assessment showed that …
Number of subjects included and excluded	Number of subjects included and excluded, and the reason for exclusion	Essential	Essential	Data was acquired in 52 subjects, of which 1 was excluded due to ecg gating issues and 3 due to insufficient data quality
Illustrations	Insightful illustrations and movies including annotations, color scaling and descriptions, clarifying which particle traces are used	Essential	Essential	Streamline visualization of aortic flow (movie in supplementary material)
Discussion				
Limitations	Discussion of the effect of uncommon or varying acquisition settings	Essential, if applicable	Essential, if applicable	Spatial resolution varied between the subject, but not between the subgroups. This is expected to
References				
Reference list	References to original work	Essential	Essential	

### Method-data processing

Data processing can be performed by several commercial CE- and FDA approved software packages, while in-house developed tools enable techniques for research. Open-source software solutions facilitate reproducible research, and the availability of such tools should be clarified in publications. For commercially available as well as open-source software, the software release version should be detailed.

### Method-quantification

After processing the data, hemodynamic parameters can be extracted from the velocity fields. A range of metrics can be derived, each with relevance depending on the specific application. Thus, a detailed description of the analysis methodology should be provided so that a similar analysis could be performed at other centers.

### Methods-statistics

Clinical diagnosis and/or outcome studies need to be designed with an adequate sample size resulting from a power analysis. The methods section should contain a statistics paragraph describing all used statistical methods appropriate for the study size.

### Results

The results section should include the number of included and excluded subjects, as well as the reason for exclusion, the results of the data quality assurance assessment, at least including a within-dataset validation (see section on "Quality assurance and validation advice in the research setting" and for clinical use), and inter- and intra-reader agreement for a subset of data (or referenced to previous publication with the same technique in the same setting) should be included. Given the variety in algorithms and their performance, it is important to mention whether velocity aliasing was present and have an estimate of how well the velocity fields have been corrected.

Besides the quantitative results, preferably shown in tables, well-made illustrations should be added of typical as well as extreme findings or participants. Movies should be included as supplementary material, if allowed by the publisher, to illustrate the behavior over the cardiac cycle. The anatomy should be well-annotated, preferably in combination with segmentation. Color bars should be included for quantitative parameters. If particle traces were used in the visualization, it should be clarified which particle traces were used (e.g., pathlines, streamlines).

### Discussion

If acquisition settings are significantly different from these and previous recommendations [1] or vary between participants, this should be mentioned in the limitation section, and it should be discussed how this might affect the results.

### References

References to methods and techniques should reference the original work.

These recommendation standards will contribute to consistently high-quality publications, will improve the review process, and allow for easier comparison of different publications. We would therefore like to stress the importance of following these standards.

## Overcoming limitations and future considerations

4D Flow CMR is becoming more widely used in medical centers with the technical and clinical capabilities to incorporate its use into standard-of-care protocols for heart valve, aortic, pulmonary, and congenital heart disease. However, several challenges remain to achieve widespread adoption and application of 4D Flow CMR remain. This includes limited velocity dynamic range due to a single user-selected VENC, long and unpredictable scan times, data storage of large datasets especially in clinical work flows as well as manual and time-consuming data processing (which can affect user confidence). Below we discuss several promising new developments which are ongoing to overcome these limitations.

### Velocity dynamic range

Acceleration techniques enable reductions in acquisition time [55, 68, 96–109] or acquisition of additional data within the same total acquisition time. In particular, acceleration enables the acquisition of additional data to reduce the dynamic range issues associated with velocity encoding. Evaluation of altered cardiovascular hemodynamics often requires measurement of flow across a wide range of velocities, e.g., high-velocity flow jets (up to 400–600 cm/s) with adjacent regions of low circulating or venous flows (as low as 10 cm/s). Commonly available 4D Flow CMR techniques measure blood flow velocity based on a single pre-defined VENC, but acceleration techniques have enabled 4D Flow CMR with dual- or multi-VENC velocity encoding [110–116], i.e., acquisition of both low- and high-VENC data within a single scan. Multi-VENC reconstruction can generate 4D Flow CMR data with the favorable VNR of a low-VENC acquisition but without velocity aliasing. In addition to multi-VENC acquisitions, initial deep learning-based studies have demonstrated the potential of using physics-informed neural networks to reduce noise, enhance resolution and automatically unwrap aliased velocity values in 4D Flow CMR velocity data [117–119].

### Respiratory and cardiac self-gating

4D Flow CMR techniques are also being developed that permit respiratory and cardiac self-gating and thereby simplify and streamline acquisition for optimized clinical workflows. Respiratory self-gating eliminates the need for respiratory navigators and can be achieved by repeatedly acquiring a central k-space line that corresponds to a projection of the image volume in the feet-to-head direction [85]. This has recently been incorporated in so-called five-dimensional “5D” and extra-dimensional “XD” Flow CMR which also permit the analysis of respiratory-driven changes in cardiovascular hemodynamics [120–124]. Cardiac self-gating can be achieved with similar principles as respiratory self-gating and has recently been incorporated in 4D Flow CMR [122, 125]. Fully self-gated 5D free-running approaches [122, 126] that exploit compressed sensing reconstruction remove the need for respiratory navigators, have constant scan time, and are independent of the patient’s breathing pattern or heart-rate, which makes it particularly well-suited to be integrated as part of a clinical protocol while scan planning is much facilitated. However, at this juncture, reconstruction times are prohibitive for clinical use.

### Accelerated data processing workflows

Current 4D Flow CMR data analysis workflows are often non-standardized and time-consuming, thus limiting reproducibility and clinical translation. Addressing these limitations will require the development of efficient image analysis strategies with minimal user dependence. This is becoming feasible with advances in image processing techniques. For example, automated segmentation of the aorta and pulmonary artery has been demonstrated with atlas-based as well as deep learning-based methods [127–129]. In addition to segmentation, machine learning has the potential to speed up and automate image processing tasks such as background phase offset correction, although work in this area is still in an early stage and unpublished. Another time-consuming task for which machine learning has demonstrated impressive results is the reconstruction of highly undersampled 4D Flow CMR images from raw data in less than 1 min [130].

## Summary/conclusion

4D Flow CMR has moved from “pretty pictures” to providing robust flow quantification in clinical practice. 4D Flow CMR has greatly benefitted from the advances in CMR acceleration making it feasible for clinical use. The worldwide 4D Flow CMR community has grown exponentially since the last consensus statement. 4D Flow CMR is no longer just a tool for researchers but for clinicians. This consensus statement aims to help clinicians initiate a 4D Flow CMR program in their institutions. Furthermore, it aims to set standards for both clinical and research settings to assure consistent high-quality 4D Flow CMR output.

## Data Availability

Not applicable.

## Appendix 1

### 4D Flow CMR sequence options

CMR is a relatively time-consuming medical imaging modality. Standard 4D Flow CMR requires particularly long scan times as it requires motion encoding gradients in three directions, respiratory motion suppression and the acquisition of multiple cardiac phases to capture hemodynamics throughout the cardiac cycle. Accelerated acquisition strategies are important for both research, and clinical practice and a vast variety of 4D Flow CMR sequences are now available. This section reviews important considerations when choosing a 4D Flow CMR sequence for both research and clinical applications.

### Flow encoding

Multiple approaches are in use that can achieve three-directional flow encoding in phase-contrast CMR [131]. The most straightforward method for three-directional motion encoding is the simple 4-point method which employs three acquisitions with motion encoding gradients consecutively applied in three orthogonal directions and one additional velocity insensitive reference acquisition to remove phase from other sources. In addition to the velocity vector, this type of asymmetric velocity encoding scheme enables measurements of turbulent kinetic energy [131–133]. Alternatively, a symmetric scheme with a reference acquisition that is motion encoded along all three axes and three acquisitions in which the polarity of the velocity encoding gradient along one axis is switched per acquisition achieves the same net velocity encoding with smaller velocity encoding gradients and, hence, results in shorter echo time (TE), shorter repetition time (TR), and potentially also reduced eddy current effects. Another alternative that uses 4 acquisitions is the so-called Hadamard scheme, in which the polarity of the velocity encoding gradient along two axes are switched per acquisition and all four acquisitions are combined to reconstruct velocity in a given direction. It should be noted that the velocity aliasing pattern for Hadamard and other multi-directional velocity encoding schemes is complex and requires additional processing steps to correct for [131]. Clinical users need to be aware that commercial softwares do not support phase unwrapping for Hadamard velocity encoding sequences.

There are advantages of acquiring additional motion encodes beyond standard 4-point encoding, but they come at the expense of prolonged scan times. For example, the use of 5-point encoding demonstrated an increase in VNR by 25% with increases in scan time [115]. Furthermore, dual or multi VENC acquisitions in which all three velocity encoded acquisitions are repeated with two or more VENC settings also target an improvement of VNR by combining the data acquired with different VENC using automatic phase-unwrapping by means of the high VENC data [110, 111, 114]. This may be useful in clinical applications where the accuracy of both low and high-velocity measures are of importance. The broad dynamic range obtained by this approach can also be advantageous for concurrent velocity and turbulent kinetic energy measurements. Finally, schemes with motion encoding along with a minimum number of six non-collinear axes, such as six-directional icosahedral (ICOSA6) [134], enables the quantification of all components of the Reynolds stress tensor. For clinical application a sequence based on simple four-point encoding currently remains favorable.

### k-space sampling

The acquisition scheme in 4D Flow CMR is typically based on the robust and well understood so-called spin-warp or Cartesian trajectory. Unfortunately, scan times are long because only a single line of k-space is acquired for each radio-frequency excitation. Partial k-space acquisitions in the phase- and frequency-encoding directions can reduce scan time or increase temporal resolution and reduce certain artefacts in Cartesian 4D Flow CMR. For example, partial k-space acquisitions in the frequency-encoding directions, termed partial Fourier or fractional echo acquisition, reduce TE and intravoxel dephasing that can occur in complex and turbulent flows.

Non-Cartesian sampling patterns have some compelling properties, particularly for accelerated imaging, as well as disadvantages. Radial trajectories [135] reduce apparent motion artefacts and enables accelerated imaging through radial undersampling, retrospective gating to the cardiac and respiratory cycle [122, 136], physiological self-gating [122, 137], advanced motion correction, and short TE imaging with center out trajectories [138, 139]. Spiral sampling trajectories [140] also allow for short TE imaging and undersampling. In addition, they enable long readouts for improved scan efficiency when compared to radial sampling. Both, radial and spiral trajectories are more sensitive to trajectory errors and artefacts from off-resonance effects compared to Cartesian trajectories and require computationally more demanding reconstruction engines.

### Accelerated imaging

Several approaches can be used to reduce scan time in 4D Flow CMR, at the cost of image quality and often-increased reconstruction time.

With echo planar imaging [83, 98, 99], multiple lines in k-space are acquired after a single excitation, thereby substantially increasing the data acquisition efficiency. Multi-echo acquisitions can also be used with radial 4D Flow CMR acquisitions [100] while a spiral trajectory achieves similar gains in efficiency by simply lengthening its single readout window. These approaches are ultimately limited by the irrecoverable T2* decay and phase disruptions of the signal during extended readouts. Echo planar imaging with a modest EPI factor of up to 5 appears useful to speed up 4D Flow CMR data acquisition in regions of normal cardiac blood flow and especially when the acquisition volume can be planned in such a way, that anticipated main flow in readout direction can be avoided [99, 141–143]. We therefore advise against the use of echo planar imaging for high flow velocity in anatomies where the main flow direction can change with respect to the gradient directions, such as in the ascending aorta and aortic arch.

The use of receiver coil arrays enables accelerated acquisitions with parallel imaging techniques [101–103]. Parallel imaging has found widespread adoption to reduce scan times for velocity encoded CMR [104] as well as other cardiovascular and generic CMR applications with sufficient SNR and can be used with Cartesian and Non-Cartesian trajectories.

Acceleration approaches that exploit spatial and temporal redundancy, such as k-t BLAST (Broad-use Linear Acquisition Speed-up Technique) and k-t SENSE (SENSitivity Encoding) [105] were quickly adapted to phase-contrast CMR [106, 107, 144]. More advanced derivatives such as k-t generalized autocalibrating partially parallel acquisition (GRAPPA) [145, 146], parallel CMR with extended and averaged k-t generalized autocalibrating partially parallel acquisition (PEAK GRAPPA) [145], and kt principal component analysis (kt-PCA) [147] have been used to highly accelerate 4D Flow CMR, with less residual temporal smoothing and underestimation of peak velocities and flow [148]. Our experience has been that an acceleration factor of R = 3 is approaching the limit for bulk flow measurements with SENSE.

Compressed sensing (CS) is another approach that utilizes data undersampling with a constrained reconstruction for faster imaging [108] and is now used frequently to accelerate 4D Flow CMR [70, 149]. Radial [150] and spiral [96] acquisitions are well suited for CS as the non-Cartesian acquisitions allow for more flexibility in the sampling pattern and better promote incoherent undersampling artefacts achieved with pseudo-random sampling. Local low-rank reconstructions push the envelope even further [109] in reducing scan times or for generating datasets with multiple velocity encodes [151] or ultrahigh temporal resolutions [152]. Similar to kt acceleration methods, potential systematic underestimation of peak velocities and flow with CS need to be considered when interpreting the results [153]. Finally, deep learning may serve as an alternative to traditional reconstructions and has shown promise [130].

### Respiratory motion suppression

In cardiovascular 4D Flow CMR, different respiratory motion suppression methods can be used to minimize breathing artefacts. Diaphragmatic respiratory navigators use a separate CMR acquisition once per heartbeat to follow the motion of the lung-liver interface. That signal is analyzed in real-time and used to accept or reject data based on a predefined acceptance window [154–156]. While navigators mitigate respiratory motion artifact, they can result in highly variable and prolonged scan times which can be minimized using a smaller window for the center of k-space [45, 157]. Traditional respiratory navigator temporal sampling is limited as only 1–2 respiratory positions are sampled per cardiac cycle. Consequently, inherent gating techniques, including self-gating, which typically tracks respiratory motion using the center of k-space or frequent 1D projections in the direction of diaphragmatic motion, have gained traction in recent 4D Flow CMR sequence development [55, 123, 125, 158–163]. Alternatively, optical tracking or respiratory bellows that continuously track abdominal motion offer motion tracking with high temporal resolution without interfering with the CMR signal [164]. However, shallow abdominal breathing can cause drifts and errors in motion tracking.

Respiratory ordered phase encoding, which selectively samples different parts of k-space based on respiratory position, has been adopted by multiple vendors to improve scan efficiency and image quality [40–42]. Recently, CS and motion correction have been combined to improve scan efficiency by including all data throughout the respiratory cycle with success in pediatric patients [55]. It is also worth noting that 4D Flow CMR without respiratory gating has demonstrated reasonably accurate intracardiac and vascular flow quantification in the chest with reduced scan times [148, 165, 166].

## Abbreviations

4D Flow CMR: 4D flow cardiovascular magnetic resonance
CS: Compressed sensing
ECG: Electrocardiogram
FDA: United States Food and Drug Administration
HR: Heart rate
KE: Kinetic energy
LGE: Late gadolinium enhancement
LV: Left ventricle/left ventricular
MIP: Maximum velocity projection
SAR: Specific absorption rate
SNR: Signal-to-noise ratio
SV: Stroke volume
TE: Echo time
TR: Repetition time
VENC: Velocity encoding limit
VNR: Velocity-to-noise ratio

## Declarations

### Peer review disclaimer

This work represents an official publication of the SCMR and was approved by the SCMR Executive Committee. It did not undergo *JCMR* peer-review.
